# Optimizing Pulse Frequency in Electroconvulsive Therapy

**DOI:** 10.1192/j.eurpsy.2025.760

**Published:** 2025-08-26

**Authors:** J. C. Hall, R. Grace, B. Carr

**Affiliations:** 1 College of Medicine; 2 Department of Psychiatry, University of Florida, Gainesville, United States

## Abstract

**Introduction:**

Effective seizure induction with minimal adverse effects in Electroconvulsive Therapy (ECT) are influenced by administered electrical pulse frequency. Optimized pulse frequency is key to ensure therapeutic efficacy and reduce side effects.

**Objectives:**

This study examines electrical pulse frequency impact on neuronal excitability and seizure quality in ECT, guided by chronaxie and refractory periods.

**Methods:**

A comprehensive literature review was conducted to assess neurophysiological properties affected by ECT and how different frequencies influence treatment outcomes.

**Results:**

Neurons fire action potentials when membrane potentials reach a -55 mV threshold. Lower frequencies (20-32 Hz) balance depolarization and repolarization to trigger seizures without excessive neuronal firing. The neuronal absolute refractory period is 1-2 msec, and the relative refractory period is 2-4 msec. Lower frequencies optimize repolarization recovery.

ECT is clinically administered at 20-70 Hz. Studies show 20-32 Hz is effective at triggering seizures with optimal treatment outcomes. Lower Hz also minimizes tissue damage from reduced power. The total charge delivered is affected by current amplitude, pulse width, frequency, and train duration. Shorter pulse widths (0.3 ms) reduce total energy and minimize tissue heating.

The neuronal soma is sensitive to electrical stimulation. Chronaxie is the minimum time that an electric current is applied to stimulate a neuron. Chronaxie is 0.2-0.3 msec. Aligning pulse frequencies with these values ensures stimulation with reduced adverse effects. The soma exhibits a lower spike threshold and shorter refractory period when facing prolonged steady depolarization, making it highly sensitive to pulse frequencies that align with its chronaxie values.

In contrast, axons have a higher density of voltage-gated Na+ channels that allow quicker recovery and shorter refractory periods. This high density enables axons to rapidly transmit action potentials, facilitating efficient neuronal signal propagation. Shorter axonal refractory period means they handle higher frequencies more effectively, but optimizing the overall frequency for ECT must balance the excitability of both the soma and axons.

Studies indicate that frequencies around 20-32 Hz are effective in initiating convulsive activity, aligning well with the end of the stimulus train. Frequencies over 50 Hz may suppress ictal activity and be inefficient in seizure induction due to “stimulus crowding,” with neurons stimulated during their absolute refractory period.

**Conclusions:**

Optimizing ECT pulse frequency is vital to balance therapeutic efficacy and safety. Fine-tuning ECT’s electrical parameters enhances patient outcomes. Lower frequencies (20-32 Hz) are more effective to induce seizures and minimize adverse effects. 20-70 Hz in ECT is most clinically used, and lower end Hz could optimize results. Further frequency range research could lead to improved ECT protocols.

**Disclosure of Interest:**

None Declared

